# Digital 3D exoscope is an effective tool for the surgery of falx and parasagittal meningiomas

**DOI:** 10.1007/s00701-024-06419-x

**Published:** 2025-01-08

**Authors:** Ville Vasankari, Sergio Garcia, Hrvoje Baric, Mika Niemelä, Martin Lehecka

**Affiliations:** 1 Department of Neurosurgery, Helsinki University Hospital and University of Helsinki, P.O. Box 266, FI- 00029 Helsinki, Finland; 2 Department of Neurosurgery, Clinical Hospital Center Zagreb, Zagreb, Croatia

**Keywords:** Exoscope, Meningioma, Microscope, Neurosurgery, Resection

## Abstract

**Purpose:**

Digital 3D exoscopes are promising tools for microneurosurgery. The results of exoscope-assisted resection of intracranial meningiomas have only been addressed in few case reports. We retrospectively compared the results of exoscope and microscope-assisted surgery of falx and parasagittal meningiomas.

**Methods:**

We included all consecutive adult patients (*n* = 36) with falx or parasagittal meningioma who were operated with curative intention during an 8-year period by one senior neurosurgeon. The operations were performed either with a surgical microscope (*n* = 16; Zeiss Kinevo or Pentero 900) or a digital 3D exoscope (*n* = 20, Aesculap Aeos). We reviewed the pre- and postoperative radiological images, clinical examinations and surgical reports to assess clinical outcomes and complications. We also analyzed surgical videos.

**Results:**

Gross-total resection (Simpson grade I-II) was achieved in approximately 90% of the patients in both groups (89% in exoscope and 92% in microscope group). The duration of the operation was slightly longer (117 vs. 88 min) in the exoscope group. Surgical outcomes were comparable, despite there being larger tumors (median diameter 53 vs. 38 mm) with higher grades (WHO Grade 2–3: 45% vs. 19%) in the exoscope group. Transient postoperative complications were more frequent in the exoscope group (40 vs. 25%) mainly related to the larger tumor size.

**Conclusion:**

The digital 3D exoscope is an effective tool for performing surgery on falx and parasagittal meningiomas. The extent of removal, clinical results and complications seem to be comparable to surgical microscope even in large tumors. Larger prospective studies are required to confirm this result.

**Supplementary information:**

The online version contains supplementary material available at 10.1007/s00701-024-06419-x.

## Introduction

Falx and parasagittal meningiomas account for approximately 20% of intracranial meningiomas [10, 16]. Meningiomas in these locations are often larger and classified with higher WHO grades than e.g., skull base meningiomas [16, 11]. Management includes observation, surgery, and radiosurgery. Microsurgical resection, which may involve the removal of some adjacent structures (such as dura and bone), is considered the therapeutic gold standard.

Digital 3D exoscope has been recently introduced as an alternative tool to surgical microscopes in the intraoperative illumination and magnification for microneurosurgery [14, 19]. Exoscopes are equipped with high-definition digital 3D camera attached to a robotic arm and controlled e.g., with foot pedal [8]. Benefits of exoscopes, such as improved ergonomics and superior magnification, have been reported in laboratory studies [7, 9]. In retrospective clinical series on complex neurosurgical pathologies (e.g. cerebral aneurysms, vestibular schwannomas, spinal dural arteriovenous fistulas, intradural extramedullary spinal tumours), the use of exoscopes have led to comparable results to microscopes [1, 2, 18, 21]. However, on exoscopic intracranial meningioma surgery there are only few case reports or series [3, 4].

In this study, we compare single neurosurgeon’s results of exoscope and microscope-assisted surgery of falx and parasagittal meningiomas in a consecutive retrospective series. Our objective was to compare the devices in surgeries where the setting and positioning are complex. Our primary hypothesis is that the exoscope is effective tool in surgery of these lesions with respect to clinical outcome, postoperative complications and extent of resection.

## Methods and materials

### Study design and setting

We included all adult patients (*n* = 36) surgically treated for parasagittal (*n* = 13) or falx (*n* = 22) meningiomas (FM) operated by one neurosurgeon (ML) between January 2016 to January 2024. Information on the attachment (parasagittal vs. falx) of one meningioma was not available. The surgeries were performed at a tertiary neurosurgical referral center using either a surgical microscope (*n* = 16; Zeiss Kinevo^®^ or Pentero^®^) or a digital 3D exoscope (*n* = 20; Aesculap Aeos^®^). The transition from surgical microscope to exoscope took place gradually during 2019–2020. Since January 2021, all the surgeries were done with an exoscope. The research review board of Helsinki University Hospital approved the study, which we designed according to the Declaration of Helsinki.

### Patients

Patients were included in the current study according to the following eligibility criteria:

(i) age over 18;
(ii) histologically confirmed meningioma
(iii) falx meningioma - i.e. arising from the *falx* and completely concealed by the overlying cortex, or parasagittal - i.e. meningiomas based at the junction of the falx and the convexity dura, which grow into at least, one wall of the superior sagittal sinus.
(iv) Intended gross total resection of the tumour.

### Variables and data sources

#### Patient data

Patients’ data regarding their preoperative clinical and radiological examinations, surgical reports, intraoperative videos, postoperative course, and clinical and radiological examination at follow up visit was reviewed.

#### Videos

The intraoperative videos were available for 17 (85%) and 12 (75%) of the patients in the exoscope and microscope groups, respectively. Surgical videos were retrieved from the institutional repository and reviewed by two independent neurosurgeons. Various surgical parameters were assessed, including: (i) zoom adjustments; (ii) focus adjustments; (iii) angular movement of the optics (excluding translational movements); (iv) tumor visualization quality; (v) superior sagittal sinus ligation; (vi) tumor consistency (soft, medium, hard/fibrotic); (vii) adherence to the brain (clear cleavage plane, slightly adherent and adherent with poor cleavage plane); (viii) time from dural incision to closure, (ix) perceived video quality. The perceived video quality was evaluated using the Mean Opinion Score, which rated it as excellent, acceptable, or poor based on the observer’s general qualitative opinion of image sharpness, color contrast, and the ability to distinguish different structures [13].

### Surgical technique

**Fig. 1:**
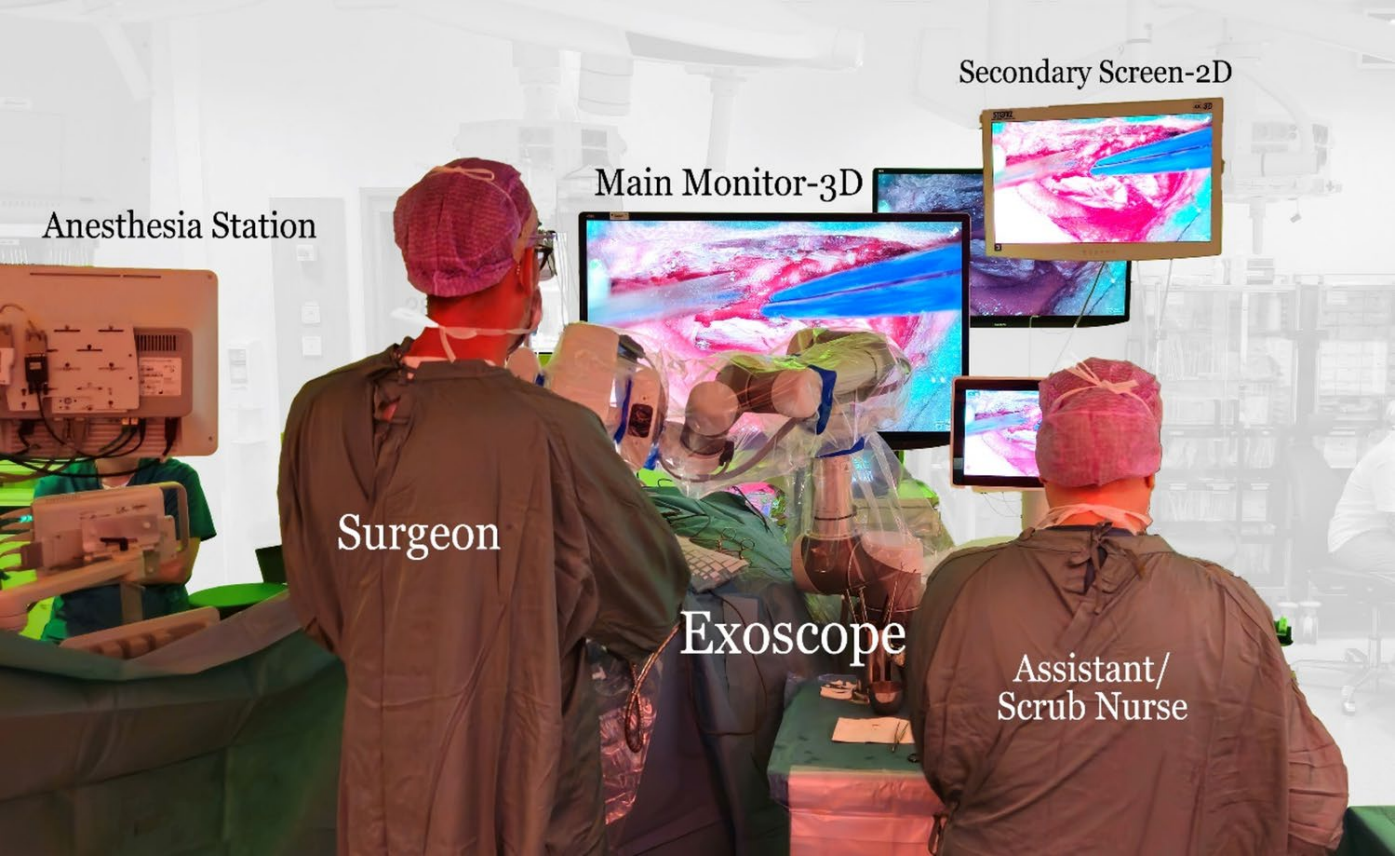
The setup for the exoscope-assisted resection of falx and parasagittal meningiomas

Neuronavigation was used in all cases to plan the bony opening. Depending on the location of the tumor the surgeries were done either in supine (frontal tumors) or lateral park-bench position (postero-frontal and parietal/occipital tumors). In lateral position for parasagittal meningiomas tumor side was up, for falx meningiomas tumor side was down for gravity retraction. Head was clamped in a Sugita head frame. Armrest was used to ease the stabilization of the surgeon’s arms. One or two burr holes were placed on top of the SSS and craniotomy was customized according to tumor’s location and size. Dura was opened in a “U-Shaped” fashion with its base towards the SSS. The magnification device was usually introduced following the dural opening (sometimes already for opening of the dura) and used until the closure in all cases. Microsurgical technique was employed to remove the tumor: (1) devascularization of the main tumor feeders (either dura or falx) with bipolar, (2) initial extracapsular dissection to expose the tumor, (3) tumor debulking with or without ultrasonic aspirator depending on tumor’s consistency, and (4) tumor capsule dissection and removal. Tumor was circumdissected with a combination of sharp and blunt dissection. If involved by the tumor, dura was removed and substituted by a vascularized galea graft. In cases with tumor infiltration into the superior sagittal sinus, most often this small remnant was left in place and later followed. The setup for the exoscope-assisted resection of falx and parasagittal meningiomas is illustrated in the Fig. 1.

### Statistical analysis

Data collection was performed using Microsoft Excel (Microsoft, Redmond, WA, USA; version 16.16.4). Statistical analyses were conducted in IBM SPSS^®^ Statistics for Windows, version XX (IBM Corp., Armonk, N.Y., USA). The distribution of quantitative variables across groups was examined using independent-samples t-test. Qualitative variables distribution was tested using the Fisher´s exact test. Level of significance was set at *P* < 0.05 and all tests were two-sided.

## Results

### Patients and meningiomas

A total of 36 patients with median age of 68 years (range 38–82) were included in this study (Tables 1 and 2). The most common indication for surgery was symptomatic meningioma in both groups (72%), followed by the asymptomatic tumours with growth pattern in the radiological follow-up (19%). However, the proportion of asymptomatic patients was higher in the microscope group (31% vs. 20%), while the proportion of preoperatively dependent patients was higher in the exoscope group (25% vs. 6%). The proportion of parasagittal meningiomas was higher (45% vs. 27%) and the tumors were larger (median diameter 53 vs. 38 mm) in the exoscope group. The proportion of WHO Grade 2–3 meningiomas was higher in the exoscope group (45% vs. 19%). The meningiomas of the exoscope group were larger, more often located in parasagittal area and more commonly atypical or anaplastic.

**Table 1:** The preoperative patient evaluation, meningioma characteristics, and surgical outcomes among patients undergoing exoscope- or microscope-assisted resection of falx and parasagittal meningiomas

	Exoscope group (*n* = 20)	Microscope group (*n* = 16)	Significance
Patients and preoperative evaluation			
Age (years): median (Range)	68 (40–78)	66 (38–82)	0.43
Women: *n* (%)	14 (70%)	8 (50%)	0.31
Preoperative condition: *n* (%)			
-Independent at home -Supported at home -Hospital ward	15 (75%) 2 (10%) 3 (15%)	15 (94%) 0 1 (6%)	0.43
Indication for surgery: *n* (%)			
-Symptomatic meningioma -Growth in radiological follow-up -Large asymptomatic meningioma	15 (75%) 3 (15%) 2 (10%)	11 (69%) 4 (25%) 1 (6%)	0.86
Preoperative symptoms			
-Focal neurological deficit -Epilepsy -Asymptomatic -Lowered consciousness	9 (45%) 6 (30%) 4 (20%) 1 (5%)	7 (44%) 3 (19%) 5 (31%) 1 (6%)	0.66
Meningioma characteristics			
Origin: *n* (%)			
-Falx -Parasagittal	11 (55%) 9 (45%)	11 (73%) 4 (27%)	0.31
Diameter (mm): Median (Range)	53 (17–74)	38 (23–63)	0.01
Location: *n* (%)			
-Frontal -Parietal -Occipital	17 (85%) 3 (15%) 0	12 (75%) 3 (19%) 1 (6%)	0.65
Surgery			
Position of patient: *n* (%)			
-Supine -Park bench -Prone	14 (70%) 6 (30%) 0	9 (56%) 5 (31%) 2 (13%)	0.35
Bifalcine resection of the meningioma: *n* (%)	10 (50%)	6 (38%)	0.52
Sagittal superior sinus: *n* (%)			
-Intraoperative bleeding -Ligation	0 0	1 (6%) 0	
Bridging veins coagulated: n (%)	3 (15%)	1 (6%)	n/a
Intraoperative bleeding (ml): median (range)	500 (120–1200)	120 (50–500)	< 0.001
Histopathology report			
WHO Grade: *n* (%)			
−1 −2 −3	11 (55%) 8 (40%) 1 (5%)	13 (81%) 3 (19%) 0	0.16
MIB-1 -index: Median (range)	7 (4–20)	8 (5–15)	0.88

**Table 2:** The characteristics and outcomes of patients scheduled for falx or parasagittal meningioma surgery

Patient	Device (microscope vs. exoscope)	Age (years)	Meningioma diameter (mm)	Origin	Intradural operation time (min)	WHO Grade	Residual meningioma diameter (mm)
1	Microscope	70	38	Falx	117	2	0
2	Microscope	71	58	Parasagittal	233	2	n/a
3	Microscope	82	42	Falx	134	1	n/a
4	Microscope	72	29	Falx	n/a	1	0
5	Microscope	62	63	Parasagittal	n/a	1	0
6	Microscope	44	38	Falx	67	1	0
7	Microscope	70	31	Parasagittal	129	1	26
8	Microscope	78	39	n/a	n/a	1	0
9	Microscope	64	47	Falx	n/a	1	0
10	Microscope	59	37	Falx	n/a	2	0
11	Microscope	72	38	Falx	86	1	n/a
12	Microscope	60	24	Falx	65	1	0
13	Microscope	59	23	Falx	45	1	0
14	Microscope	54	32	Falx	42	1	0
15	Microscope	68	38	Parasagittal	88	1	0
16	Exoscope	69	28	Falx	126	1	0
17	Microscope	38	62	Falx	153	1	0
18	Exoscope	78	55	Falx	164	1	0
19	Exoscope	64	40	Falx	82	1	0
20	Exoscope	74	67	Parasagittal	54	3	n/a
21	Exoscope	74	33	Falx	93	2	0
22	Exoscope	53	48	Falx	n/a	1	0
23	Exoscope	61	63	Falx	208	1	0
24	Exoscope	48	57	Falx	119	2	0
25	Exoscope	67	74	Falx	114	2	0
26	Exoscope	55	69	Parasagittal	125	2	0
27	Exoscope	78	44	Falx	127	1	n/a
28	Exoscope	55	17	Parasagittal	n/a	1	0
29	Exoscope	40	67	Parasagittal	n/a	2	0
30	Exoscope	76	52	Parasagittal	130	2	0
31	Exoscope	75	64	Parasagittal	177	2	0
32	Exoscope	67	70	Parasagittal	104	1	0
33	Exoscope	72	50	Falx	n/a	1	10
34	Exoscope	77	46	Parasagittal	77	2	5
35	Exoscope	70	50	Parasagittal	86	1	0
36	Exoscope	40	53	Falx	37	1	0

### Surgical treatment and surgical complications

Surgical details are presented in Table 1. There were no major bleedings from the SSS in any of the cases. In one case (microscope group) a venous air embolism occurred without postoperative consequences. This incident resulted in a shift towards using lateral position later in the series, especially for tumors located in fronto-parietal and parietal area. Bridging veins had to be sacrificed in three (15%) and one (6%) patients in the exoscope and microscope groups, respectively. Two (50%) of these patients, both in the exoscope group, developed a new or worsened neurological deficit postoperatively. One of them had frontal falx meningioma with diameter of 63 mm and the other parietal parasagittal meningioma with diameter of 52 mm. The decisions to sacrifice the bridging veins were related to big and challenging tumors rather than differences in the magnification devices. The intraoperative blood loss was higher (500 vs. 120 ml) and the duration of the operation longer (117 vs. 88 min) in the exoscope group. The median duration of the exoscope-assisted surgeries decreased by 24 min in the latter half of the data collection. Overall, considering larger tumors and higher WHO grades in the exoscope group, surgical outcomes were comparable between the two analyzed devices.

### Intraoperative video analysis

Results of the surgical video analysis are presented in Table 3. The exoscope was tilted (median 42 vs. 38 times) and zoomed (median 20 vs. 12 times) more often than microscope. The exoscope was more often out of focus (9 vs. 3 times) during each surgery compared to the microscope. The quality of the intraoperative recordings was rated good more often in the exoscope group (82% vs. 67%). Visualization of the surgical angle of approach was optimal in all exoscope cases, but suboptimal in one patient in the microscope group.

**Table 3:** The analysis of intraoperative videos of exoscope- and microscope-assisted resection of falx or parasagittal meningiomas

	Exoscope group (*n* = 20)	Microscope group (*n* = 16)	Significance
Intraoperative videos			
Zooming (*n*): median (range)			
-In -Out	12 (2–22) 8 (1–18)	7 (3–20) 5 (1–15)	0.10 0.14
Out of focus (*n*): median (range)	9 (0–60)	3 (0–47)	0.65
Tilting (angular movement) (*n*): median (range)	42 (10–115)	28 (10–95)	0.18
Video quality: *n* (%)			
-Good -Mediocre -Poor	14 (82%) 2 (12%) 1 (6%)	8 (67%) 3 (25%) 1 (8%)	0.56
Consistency of meningioma: *n* (%)			
-Soft (Only suction used) -Medium (+ ultrasonic aspirator) -Hard/fibrotic (scissors or rongeur)	10 (59%) 6 (35%) 1 (6%)	5 (42%) 2 (17%) 5 (42%)	0.09
Adherence to brain: *n* (%)			
-Not adherent (clear border) -Medium (easily dissected) -Adherent (challenging Dissection, infiltrating tumor)	7 (41%) 10 (59%) 0	5 (42%) 5 (42%) 2 (17%)	0.27
Time from dural incision to beginning of closure (min): Median (range)	117 (37–208)	88 (42–233)	0.66

### Postoperative complications/deficits

Postoperative treatment and postoperative complications are presented in Table 4. Only two patients, one in each group, spent longer than one day in the intensive care unit postoperatively (Table 4). Eight (40%) patients in the exoscope group and four (25%) in the microscope group suffered from any postoperative complications. The most common complication was new or worsened neurological deficit observed in four (20%) exoscope patients and two (13%) microscope patients. These deficits resolved in four out of the six patients (exoscope *n* = 3, microscope *n* = 1) by the time of a three-month follow-up visit. A bridging vein was intraoperatively coagulated and cut in two of these patients. There was only one patient with permanent new neurological deficit in each of the group. One patient in the exoscope group required re-operation due to a late postoperative wound infection. Postoperative hematomas occurred in neither group.

**Table 4:** Postoperative treatment, follow-up and complications among patients undergoing exoscope- or microscope assisted resection of falx or parasagittal meningiomas

	Exoscope group (*n* = 20)	Microscope group (*n* = 16)	Significance
Postoperative treatment			
Length of stay in ICU (*n* of days): median (range)	1 (1–3)	1 (1–2)	0.77
Length of hospital treatment^a^ (*n* of days): median (range)	5 (3–10)	4 (2–7)	0.10
Discharge: *n* (%)			
-Home directly -Rehabilitation unit	11 (55%) 9 (45%)	10 (63%) 6 (38%)	0.74
Postoperative complications			
-Total -Ischemic stroke -Wound infection -Pulmonary embolism -New epileptic symptom -New or worse focal deficit -Pneumonia	8 (40%) 1 (5%) 1 (5%) 0 1 (5%) 4 (20%) 1 (5%)	4 (25%) 1 (6%) 0 1 (6%) 0 2 (13%) 0	0.93
Re-operation due to complication *n* (%)	1 (5%)	0	
Indication for the re-operation:*n* (%)			
-Infection	1 (5%)	0	
Follow-up visit			
Evolution of preoperative symptoms:* n* (%)^b^			
-Completely resolved -Partially relieved -Same as preoperatively -Worsened -New postoperative symptom -Asymptomatic pre- and postoperatively	4 (21%) 4 (21%) 5 (26%) 2 (11%) 0 4 (21%)	1 (7%) 5 (33%) 1 (7%) 2 (13%) 1 (7%) 5 (33%)	0.42
Residence: *n* (%)^b^			
-Independent at home -Supported at home -Nursing home -Hospital ward	15 (79%) 2 (11%) 0 2 (11%)	13 (87%) 0 0 2 (13%)	0.64
Residual tumor in MRI: * n* (%) c	2 (11%)	1 (8%)	0.16

^a^Among the patients discharged from the neurosurgery ward to home directly

^b^The information is missing from one patient in the exoscope group and one in the microscope group

^c^The information is missing from two patients in the exoscope group and three in the microscope group

### Follow-up

The information on postoperative follow-up visit was available for 34 (94%) patients (Table 4). One patient in both groups was missing. Of the four patients who were not capable of independent living at the follow-up visit, three were already hospitalized preoperatively whereas only one developed a new neurological deficit postoperatively. Thirteen (68%) and 11 (73%) patients in the exoscope and microscope groups either neurologically improved or had stable condition postoperatively. The postoperative MRI images were available for 18 (90%) and 13 (81%) patients in the exoscope and microscope groups, respectively. Two (11%) patients in the exoscope group and one (8%) in the microscope group had postoperative tumor residual with volume less than 10% of the preoperative tumor. The tumor residuals located at least partially inside the superior sagittal sinus. All three patients with tumor residual were older than 70 years at the time of the surgery. The surgeon had made an intraoperative decision to purposely leave residual tumor in these patients due to challenging tumor resection, minimal risk of meningioma progression in the remaining lifetime and high risk of postoperative complications.

## Discussion

Supporting our primary hypothesis, exoscope turned out to be an effective tool for the microsurgical removal of parasagittal and falx meningiomas. Gross-total resection was accomplished in the majority of patients in both groups with similar clinical outcome. Some differences in the results such as operative time, blood loss and new transient neurological deficits were probably more related to the differences in the study groups (larger tumors, parasagittal location, higher WHO grades) than the magnification device itself. Parasagittal meningiomas are usually more involved with the bridging veins, they infiltrate the SSS more often. They also infiltrate the dura and sometimes even the bone. All these factors increase the risk for intraoperative bleeding, vascular damage and need for more extensive dural repair, all affecting the surgical time. In addition, in higher WHO grades the meningiomas cause usually more swelling in the surrounding brain and more significant symptoms as was seen in the exoscope group with larger proportion of patients hospitalized already prior to the treatment predisposing them to more postoperative complications.

Previous studies on exoscope-assisted surgery of intracranial meningiomas are scarce. Only few case-reports and small series have been published previously [3, 4, 12, 15, 17]. Authors highlighted improved visualization of the surgical field with better surgeon’s ergonomics [4, 12] while maintaining a similar safety and efficacy profile [15]. Also, our results support the idea of equivalent clinical results and risk profile of exoscopic meningioma surgery. We did not specifically look in the field of ergonomics in our current series. However, especially parieto-occipital falx meningiomas operated on park-bench position seem good targets for the exoscope-assisted surgery since the device allows extreme tilting of the camera head while maintaining ergonomical posture for the surgeon.

The rate of tumor removal and complications vary in previously published series. In a single surgeon’s series of 58 patients with parasagittal and falx meningiomas, all tumors were either completely or subtotally removed and the rate of neurological complications was low, only 5% [5]. However, the proportion of high-grade meningiomas was lower than in our study. In another series of 135 patients with falx or parasagittal meningiomas, gross-total resection was achieved in majority of the patients, especially in those without SSS invasion, while 19% of the patients developed postoperative complications [20]. In both of these series the patients were younger than in our study. The patient selection (tumor size, the proportion of parasagittal tumors, WHO Grades) probably affects the outcomes.

The different adjustments of the magnification device (tilting, adjustments of zoom and focus) to gain an optimal view to the tumor were more frequent with the exoscope than the microscope. This is probably explained by the fact that the angle of the exoscope can be more easily adjusted remotely with a foot pedal while keeping both hands in the operative field. Tilting the microscope requires the surgeon to move at least one hand away from the operative field which inevitably interrupts the workflow. In this respect one would have expected shorter operative times with the exoscope. This was not the case. The probable explanations are (a) larger tumors in the exoscope group and (b) adaptation to the exoscopic device. As this is a consecutive series, it included patients operated also during the early cases of the new magnification device. Adaptation to the controls of the exoscope takes time and practice [19]. We saw significantly shorter operative time in the latter half of the exoscopic series than in the initial half. However, there were no differences in the incidence of postoperative complications.

Exoscope provided superior intraoperative video quality compared to the microscope. This is an important feature for performance, research and teaching purposes. This finding is supported by our previous studies [6]. In addition, the exoscope magnification has been reported similar or superior when compared to the microscope in the previous studies [7, 9].

### Limitations

This study harbors some limitations. First, due to the limited study population, the statistical analysis was mostly descriptive and lacked the power for more extensive analysis. Studies with larger number of patients should be conducted to confirm our results. It should be noted that the previous studies addressing the outcomes of exoscope-assisted surgery on intracranial meningiomas are only based on small case series or reports with small sample size (e.g., *n* < 5 patients). Secondly, the senior author that performed all the cases, gained experience during the 8-year study period, which could have affected the outcomes. However, the meningiomas operated on with exoscope were more challenging (larger and more frequently high-grade) at the end of the data collection, which have balanced the effect of the learning curve. In addition, the transition from microscope to the exoscope in the middle of the data collection may also balance this effect, since it will take hundreds of hours to adjust to the new magnification device. Third, the two groups, although similar, were not equally distributed with respect to tumor origin, size and WHO grade so their risk profile was different. Fourth, due to the retrospective study design, we were not able to measure some important parameters related to the magnification devices, such as surgeon’s working ergonomics. Prospective studies are required to confirm our results. Fifth, since the operations were performed by only one surgeon, the results cannot be generalized to all the neurosurgeons. However, the surgeon had operated on almost as many cases with the two devices during his career, which makes the comparison more reliable. Most neurosurgeons have much more experience in the microscope-assisted surgery.

## Conclusions

This study demonstrates that surgical resection of parasagittal and falx meningiomas using a digital 3D exoscope is feasible and might provide similar resection rates and clinical results as a surgical microscope. Exoscope showed benefits in the intraoperative visualization allowing for better remote adjustment of zoom and viewing angle. Especially for teaching purpose, exoscope is a good choice for its better intraoperative quality. Prospective studies with larger sample size and more surgeons are required to confirm our results.

## Supplementary information

Below is the link to the electronic supplementary material.

ESM 1 (AVI 235 MB)

## Data Availability

No datasets were generated or analysed during the current study.

## References

[1] Auricchio AM, Calvanese F, Vasankari V, Raj R, Gallé CLC, Niemelä M, Lehecka M (2024) Digital exoscope versus surgical microscope in spinal dural arteriovenous fistula surgery: a comparative series. Neurosurg Focus 56(3):E13. 10.3171/2023.12.FOCUS23756 38428000 10.3171/2023.12.FOCUS23756

[2] Calvanese F, Auricchio AM, Vasankari V, Raj R, Gallè CLC, Niemelä M, Lehecka M (2024) Digital 3D exoscope is safe and effective in surgery for intradural extramedullary tumors: a comparative series. World Neurosurg 184:e1–e8. 10.1016/j.wneu.2024.01.136 38307199 10.1016/j.wneu.2024.01.136

[3] Carrasquilla A, Zgurov A, Salih M, Le C, Matsoukas S, Feng R, Schupper AJ, Hadjipanayis C (2024) Exoscopic resection of a parasagittal atypical meningioma. Neurosurg Focus Video 10(1):V8. 10.3171/2023.10.FOCVID23164 38283808 10.3171/2023.10.FOCVID23164 PMC10821652

[4] de Andrade EJ, Recinos PF (2023) Exoscopic supraorbital approach for a tuberculum sellae meningioma. Clin Neurol Neurosurg 231:107830. 10.1016/j.clineuro.2023.107830 37356198 10.1016/j.clineuro.2023.107830

[5] Eichberg DG, Casabella AM, Menaker SA, Shah AH, Komotar RJ (2020) Parasagittal and parafalcine meningiomas: integral strategy for optimizing safety and retrospective review of a single surgeon series. Br J Neurosurg 34(5):559–564. 10.1080/02688697.2019.1635988 31284785 10.1080/02688697.2019.1635988

[6] Haeren R, Hafez A, Lehecka M (2022) Visualization and maneuverability features of a robotic arm three-dimensional Exoscope and operating microscope for clipping an unruptured intracranial aneurysm: video comparison and technical evaluation. Oper Neurosurg (Hagerstown) 22(1):28–34. 10.1227/ONS.0000000000000060 34982902 10.1227/ONS.0000000000000060

[7] Hafez A, Haeren RHL, Dillmann J, Laakso A, Niemelä M, Lehecka M (2021) Comparison of operating microscope and exoscope in a highly challenging experimental setting. World Neurosurg 147:e468–e475. 10.1016/j.wneu.2020.12.093 33385603 10.1016/j.wneu.2020.12.093

[8] Hafez A, Haeren R, Huhtakangas J, Nurminen V, Niemelä M, Lehecka M (2023) 3D exoscopes in experimental microanastomosis: a comparison of different systems. Life (Basel) 13(2):584. 10.3390/life13020584 36836941 10.3390/life13020584 PMC9966143

[9] Herlan S, Marquardt JS, Hirt B, Tatagiba M, Ebner FH (2019) 3D exoscope system in neurosurgery-comparison of a standard operating microscope with a new 3D exoscope in the Cadaver Lab. Oper Neurosurg (Hagerstown) 17(5):518–524. 10.1093/ons/opz081 31140555 10.1093/ons/opz081

[10] Huntoon K, Toland AMS, Dahiya S (2020) Meningioma: a review of clinicopathological and molecular aspects. Front Oncol 23:579599. 10.3389/fonc.2020.579599 10.3389/fonc.2020.579599 PMC7645220 33194703

[11] Ketter R, Rahnenführer J, Henn W, Kim YJ, Feiden W, Steudel WI, Zang KD, Urbschat S (2008) Correspondence of tumor localization with tumor recurrence and cytogenetic progression in meningiomas. Neurosurgery 62(1):61–69. 10.1227/01.NEU.0000311062.72626.D6. (discussion 69–70) 18300892 10.1227/01.NEU.0000311062.72626.D6

[12] Kijima N, Kinoshita M, Takagaki M, Kishima H (2021) Utility of a novel exoscope, ORBEYE, in gravity-assisted brain retraction surgery for midline lesions of the brain. Surg Neurol Int 12:339. 10.25259/SNI_320_2021 34345480 10.25259/SNI_320_2021 PMC8326087

[13] Koho S, Fazeli E, Eriksson JE, Hänninen PE (2016) Image quality ranking method for microscopy. Sci Rep 6:28962. 10.1038/srep28962 27364703 10.1038/srep28962 PMC4929473

[14] Langer DJ, White TG, Schulder M, Boockvar JA, Labib M, Lawton MT (2020) Advances in intraoperative optics: a brief review of current exoscope platforms. Oper Neurosurg (Hagerstown) 19(1):84–93. 10.1093/ons/opz276 31529083 10.1093/ons/opz276

[15] Lin M, Bakhsheshian J, Strickland B, Rennert RC, Chu RM, Chaichana KL, Zada G (2020) Exoscopic resection of atrial intraventricular meningiomas using a navigation-assisted channel-based trans-sulcal approach: case series and literature review. J Clin Neurosci 71:58–65. 10.1016/j.jocn.2019.10.017 31711892 10.1016/j.jocn.2019.10.017

[16] Magill ST, Young JS, Chae R, Aghi MK, Theodosopoulos PV, McDermott MW (2018) Relationship between tumor location, size, and WHO grade in meningioma. Neurosurg Focus 44(4):E4. 10.3171/2018.1.FOCUS17752 29606048 10.3171/2018.1.FOCUS17752

[17] Price G, Schupper A, Kalagara R, Chennareddy S, He C, Zhang JY, Sudhir S, Rentzeperis F, Wanna G, Hadjipanayis C (2023) Application of the robotic-assisted digital exoscope for resection of posterior fossa tumors in adults: a Series of 45 cases. Oper Neurosurg (Hagerstown) 25(5):397–407. 10.1227/ons.0000000000000838 37523626 10.1227/ons.0000000000000838

[18] Rossmann T, Veldeman M, Nurminen V, Huhtakangas J, Niemelä M, Lehecka M (2023) 3D exoscopes are noninferior to operating microscopes in aneurysm surgery: comparative single-surgeon series of 52 consecutive cases. World Neurosurg 170:e200–e213. 10.1016/j.wneu.2022.10.106 36334715 10.1016/j.wneu.2022.10.106

[19] Silva JM, Rustemi O, Vezirska DI, Niemelä M, Lehecka M, Hafez A (2023) Taming the exoscope: a one-year prospective laboratory training study. Acta Neurochir (Wien) 165(8):2037–2044. 10.1007/s00701-023-05664-w 37369773 10.1007/s00701-023-05664-w PMC10409657

[20] Sughrue ME, Rutkowski MJ, Shangari G, Parsa AT, Berger MS, McDermott MW (2019) Results with judicious modern neurosurgical management of parasagittal and falcine meningiomas. Clin J Neurosurg 114(3):731–737. 10.3171/2010.9.JNS10646 10.3171/2010.9.JNS10646 20950085

[21] Veldeman M, Rossmann T, Huhtakangas J, Nurminen V, Eisenring C, Sinkkonen ST, Niemela M, Lehecka M (2023) Three-dimensional exoscopic versus microscopic resection of vestibular schwannomas: a comparative series. Oper Neurosurg (Hagerstown) 24(5):507–513. 10.1227/ons.0000000000000602 36715988 10.1227/ons.0000000000000602

